# Association of blood ptau‐217 with Memory Performance and *APOE* ε4 in Individuals with Subjective Cognitive Decline: insights from the BRASCODE Cohort

**DOI:** 10.1002/alz70861_108931

**Published:** 2025-12-23

**Authors:** Manuella Edler Zandoná Giordani, Barbara Loeblein Uebel, Victória Tizeli Souza, Simone Sieben da Mota, Bruno De Oliveira De Marchi, Carolina Rodrigues Formoso, Gabriela Raquel Paz Rivas, Guilherme Da Silva Carvalho, Haniel Bispo De Souza, Lucas Bastos Beltrami, Rhaná Carolina Santos, Samuel Masao Suwa, Wesley Slaviero, Ana Letícia Amorim de Albuquerque, Matheus Zschornack Strelow, Wyllians D Borelli, Giovanna Carello‐Collar, Maila Rossato Holz, Marcia L. Chaves, Eduardo R. Zimmer, Raphael Machado Castilhos

**Affiliations:** ^1^ Universidade Federal do Rio Grande do Sul, Porto Alegre, RS Brazil; ^2^ Hospital de Clínicas de Porto Alegre, Porto Alegre, RS Brazil; ^3^ Federal University of Rio Grande do Sul, Porto Alegre, Rio Grande do Sul Brazil; ^4^ Hospital de Clínicas de Porto Alegre, Porto Alegre, Rio Grande do Sul Brazil; ^5^ Universidade Federal do Rio Grande do Sul, Porto Alegre Brazil; ^6^ Universidade Federal do Rio Grande do Sul, Porto Alegre, Rio Grande do Sul Brazil; ^7^ Universidade do Vale do Rio dos Sinos, São Leopoldo, Rio Grande do Sul Brazil; ^8^ PUCRS, Porto Alegre, Rio Grande do Sul Brazil; ^9^ Hospital de Clinicas de Porto Alegre, Porto Alegre, RS Brazil; ^10^ Federal University of Rio Grande do Sul (UFRGS), Porto Alegre, RS Brazil

## Abstract

**Background:**

Subjective cognitive decline (SCD), or self‐perceived cognitive impairment, is a recognized risk factor for future cognitive decline and dementia. Analyzing Alzheimer's disease (AD) biomarkers in SCD populations could provide a means to identify preclinical AD and enable early intervention strategies. We aimed to investigate the profile of plasma phosphorylated tau 217 (*p* ‐tau217) in the Brazilian Subjective Cognitive Decline (BRASCODE) Cohort.

**Method:**

BRASCODE is an ongoing observational, prospective cohort study of cognitively unimpaired individuals over 65 years‐old with the diagnosis of SCD conducted at the Hospital de Clínicas de Porto Alegre, South Brazil. Baseline assessments included sociodemographic and clinical data collection, the Memory Complaint Scale (MCS), the Cognitive Reserve Scale (CRS), an extensive cognitive battery, apolipoprotein E (*APOE*) genotyping, and cerebrospinal fluid (CSF) and plasma Alzheimer's disease (AD) biomarker analysis. The cohort is ongoing and here we present data from the baseline evaluation.

**Result:**

A total of 148 individuals completed the baseline assessments, with 70.9% being female, with median age of 69 years and 30.2% carrying at least one *APOE* ε4 allele. *p* ‐tau217 correlated positively with age (rho=0.282, *p*<0.001) and negatively with subject‐MCS and Rey Auditory Verbal Learning Test (RAVLT) A7 and Recognition (rho= ‐0.171, *p*=0.042 and rho= ‐0.165, *p*=0.049, respectively). Compared to non‐*APOE* ε4 participants, *APOE* ε4 carriers showed higher levels of plasma *p* ‐tau217. In multiple regression analysis, *p* ‐tau217 did not significantly predict global cognition composite score (*p* =0.108). However, it was a significant predictor of A7 RAVLT results (estimate= ‐0.60, *p* <0.03), controlling for covariates.

**Conclusion:**

We observed a significant association between *p* ‐tau217, memory domain test results, and *APOE* ε4 genotype in SCD patients in the BRASCODE cohort. Ongoing follow‐up of this cohort will be crucial to elucidate further the predictive utility of AD biomarkers for cognitive change in SCD.

